# Effect of Different Types of Toothpaste on the Frictional Resistance Between Orthodontic Stainless Steel Brackets and Wires

**Published:** 2017-09

**Authors:** Tahereh Hosseinzadeh Nik, Tabassom Hooshmand, Homa Farhadifard

**Affiliations:** 1 Professor, Dental Research Center, Dentistry Research Institute, Tehran University of Medical Sciences, Tehran, Iran; Department of Orthodontics, School of Dentistry, Tehran University of Medical Sciences, Tehran, Iran; 2 Associate Professor, Research Center for Science and Technology in Medicine, Tehran University of Medical Sciences, Tehran, Iran; Department of Dental Biomaterials, School of Dentistry, Tehran University of Medical Sciences, Tehran, Iran; 3 Assistant Professor, Department of Orthodontics, School of Dentistry, Hamadan University of Medical Sciences, Hamadan, Iran

**Keywords:** Orthodontic Brackets, Orthodontic Wires, Friction, Toothpastes, Stainless Steel

## Abstract

**Objectives:**

The purpose of this study was to investigate the effect of different types of toothpaste on the frictional resistance between stainless steel brackets and archwires.

**Materials and Methods:**

Ninety stainless steel orthodontic brackets with stainless steel wires were bonded to bovine teeth and were divided into 6 groups for application of the following toothpastes: Colgate® Total® Advanced Whitening, Colgate® Total® Pro Gum Health, Colgate® Anticavity, Ortho.Kin®, and Sunstar GUM® Ortho toothpastes. No toothpaste was applied in the control group. Each group was brushed by a brushing machine with the use of the designated solution for 4.5 minutes. The frictional force was measured in a universal testing machine with a crosshead speed of 10 mm/minute over a 5-mm archwire. Data were analyzed using one-way analysis of variance (ANOVA) at the 0.05 significance level.

**Results:**

The frictional resistance values of Ortho.Kin® and GUM® Ortho toothpastes and the control group were not significantly different (P>0.05). However, there were significant differences between the frictional resistance values of Colgate® Total® Pro Gum Health and Colgate® Anticavity toothpastes with that of the control group (P<0.05). The highest and lowest frictional resistance values were related to Colgate® Total® Pro Gum Health toothpaste and the control group, respectively.

**Conclusions:**

Among the evaluated toothpastes, the orthodontic toothpastes did not increase the frictional resistance between the orthodontic stainless steel brackets and wires.

## INTRODUCTION

One of the most important elements of a successful orthodontic treatment is the control of dental caries and maintenance of a good oral hygiene [1]. Fluoride-containing commercial mouth rinses, toothpastes, and prophylactic gels are widely used to prevent dental decay and to relieve dental sensitivity in the oral cavity [2]. Regular tooth brushing removes dental stains and keeps the teeth clean. The whitening ingredients in toothpastes include chemical chelants, oxidizing agents, and particulate abrasives. Currently, the abrasives frequently used in toothpastes include precipitated silica, calcium carbonate, alumina, and a variety of calcium phosphates [3]. Myriad commercially-available dentifrices with miscellaneous ingredients are commonly used by orthodontic patients. Some of these ingredients can induce alterations in proximity to metal. These changes on the surface properties of orthodontic brackets may negatively affect the orthodontic procedure [4].

The orthodontic sliding mechanics, a technique used for closing the dental spaces, is usually performed by moving the brackets along the archwire or by sliding the wire through the brackets and molar tubes. The friction caused by the contact between the bracket and archwire is the main disadvantage affecting the sliding mechanics [5]. Friction is the force that delays or stops the movement of two materials in contact, and its direction is “tangential to the common interface of the two surfaces” [6,7]. Friction can stop the movement of the tooth to which the bracket is attached, can decrease the available force by almost 40% and may cause anchorage loss [8]. The factors that influence the friction consist of the ligation type, applied force, bracket-wire clearance, wire size and morphology, bracket dimensions, torque at the bracket-wire interface, type of movement at the bracket-wire interface, and type of bracket and wire [9].

Several studies have investigated the effects of different mouthwashes such as chlorhexidine and fluoride on the frictional resistance between orthodontic brackets and wires [5, 10–12].

Kao et al [9] immersed metal brackets and various types of orthodontic wires in the acidified phosphate fluoride (APF) prophylactic solution and investigated the frictional resistance rate. It was shown that the static frictional resistance of stainless steel, heat-activated nickel-titanium (Ni-Ti), and beta-titanium (B-Ti) alloy wires immersed in 0.2% APF solution was significantly higher than that of the wires immersed in artificial saliva [9].

Hosseinzadeh Nik et al [5] evaluated the impact of 0.2% chlorhexidine mouthwash on the surface roughness and frictional resistance between orthodontic brackets and archwires. They reported that a 1.5-hour immersion in this prophylactic agent did not have any significant influence on the archwires’ surface roughness or on the frictional resistance between stainless steel orthodontic brackets and stainless steel or Ni-Ti archwires.

Special toothpastes have been introduced to the market, specifically designed for orthodontic treatment, which can impose variable effects on orthodontic brackets and wires.

Kinoshita et al [13] investigated the impact of whitening toothpastes on the surface roughness of nanofiller-based composites. They used Colgate® Luminous White, Oral-B® 3D White and Close-Up® Diamond Attraction as whitening toothpastes, and Colgate® Total® 12 toothpaste as the control group. It was concluded that the whitening toothpastes could increase the surface roughness of the composites [13].

Barbieri et al [14] studied the influence of whitening dentifrices (Colgate® Max White and Close-Up® Extra Whitening) compared to a non-whitening dentifrice (Colgate® Total 12) on the surface roughness of commercial composites and reported that the whitening toothpastes caused higher surface roughness in the composites compared to the non-whitening type.

The effect of different types of toothpaste on the frictional resistance of orthodontic appliances has not been previously investigated. Therefore, the purpose of this study was to explore the effect of different types of toothpaste on the frictional resistance between stainless steel brackets and archwires.

## MATERIALS AND METHODS

Ninety upper central stainless-steel metal brackets (standard edgewise, Dentsply GAC International, Islandia, NY, USA) with 0.022-inch slot size were selected. 0.019×0.025-inch standard rectangular straight stainless-steel orthodontic wires (Dentsply GAC International, Islandia, NY, USA) that were cut into 8-cm pieces were used. The brackets and wires were cleaned with alcohol wipes and were observed under a stereomicroscope (Olympus, Tokyo, Japan) in order to eliminate the specimens with manufacturing defects. Bovine maxillary central incisors were collected and cleaned by immersion in 0.05% Chloramine-T solution for 7 days. The brackets were then bonded to the bovine teeth, and the orthodontic wires were tied to them using an elastomeric module (O-ring, Dentaurum intraoral elastics, Dentaurum GmbH Co., Ispringen, Germany). Next, the specimens were randomly divided into six groups for toothpaste application (n=15):

1) Colgate® Total® Advanced Whitening toothpaste
2) Colgate® Total® Pro Gum Health toothpaste
3) Colgate® Anticavity toothpaste
4) Ortho.Kin® toothpaste
5) Sunstar GUM® Ortho toothpaste
6) No toothpaste as control

The specifications of the toothpastes are provided in Table 1.

**Table 1.** Specifications of the evaluated toothpastes

**Toothpaste**	**Manufacturer**	**Lot number**	**Ingredients**	**Expiry date**
Colgate® Total® Advanced Whitening	Colgate-Palmolive Co., Ltd., China	4340	Sorbitol, Aqua, Hydrated Silica, PEG-12, Sodium Lauryl Sulfate, Aroma, Cellulose Gum, Tetrasodium Pyrophosphate, Cocamidopropyl Betaine, Sodium Fluoride, Sodium Saccharin, Hydroxypropyl Methylcellulose, Glycerin, Limonene, CI 74160, CI 74260, CI 77891, Sodium Fluoride 0.32% w/w (1450ppmF̄).	2017.12.6
Colgate® Total® Pro Gum Health	Colgate-Palmolive, Co., Ltd., China	5093	Sodium Fluoride 0.32% (1450ppmF̄), Triclosan 0.30%, Aqua, Hydrated Silica, Glycerin, Sorbitol, Sodium Lauryl Sulfate, PVM/MA Copolymer, Aroma, Cellulose Gum, Sodium Hydroxide, Propylene Glycol, Carrageenan, Sodium Saccharin, Limonene, CI 77891.	2018.4.3
Colgate® Anticavity	Colgate-Palmolive Co., Ltd., China	6050	Sodium Monofluorophosphate 0.76% (0.15% w/v fluoride ion), Dicalcium Phosphate Dihydrate, Water, Glycerin, Sodium Lauryl Sulfate, Cellulose Gum, Flavor, Tetrasodium Pyrophosphate, Sodium Saccharin.	2018.2.19
Ortho.Kin®	KIN Corp., Spain	15B05	Aqua, Sorbitol, Hydrated Silica, Glycerin, Titanium Dioxide, Aroma, Cocamidopropyl Betaine, Panthenol, Xylitol, Xanthan Gum, Peg-40, Hydrogenated Castor Oil, Sodium Fluoride, Sodium Methylparaben, Sodium Saccharin, Tocopheryl Acetate, Sodium Propylparaben, Cetylpyridinium Chloride.	2018.9.1
GUM® Ortho	Sunstar Co., Spain	J25	Aqua, Sorbitol, Hydrated Silica, Isomalt, Peg-8, Lauryl Glucoside, Aroma, Xanthan Gum, Cocamidopropyl Betaine, Panthenol, Sodium Saccharin, Sodium Fluoride, Allantoin, Sodium Methylparaben, Tocopheryl Acetate, Bisabolol, Zingiber Officinale Root Extract, Cetylpyridinium Chloride, Aloe Barbadensis Leaf Juice, CI 47005, CI 42090, Potassium Sorbate, Sodium Benzoate, Limonene.	2018.11.11

**Fig. 1:**
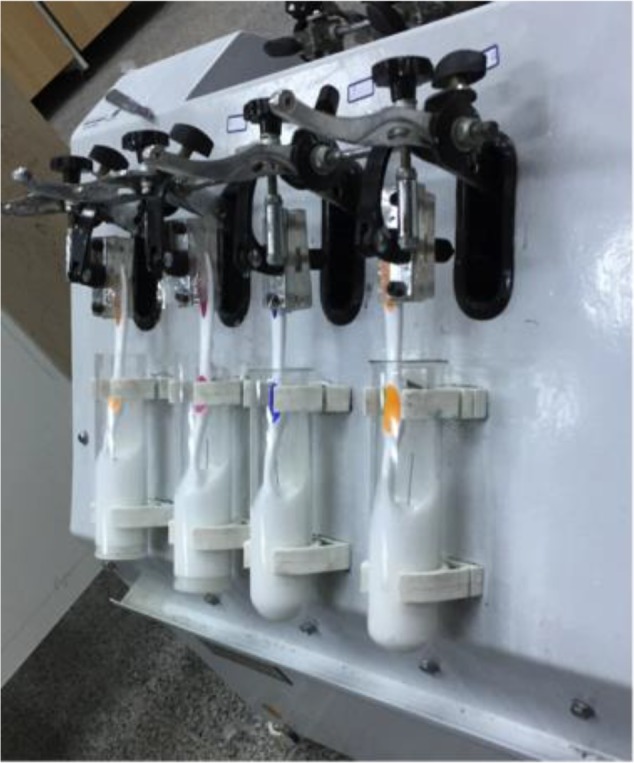
V-8 cross brushing machine

V-8 cross brushing machine (Fig. 1, Oaj Andish Spadan Co., Isfahan, Iran) was used for brushing the teeth according to the ISO 11609:2017 standard [15]. A solution containing 25g toothpaste, 20cc of modified Fusayama artificial saliva with the pH of 6.75 containing NaCl (400mg/l), KCl (400mg/l), CaCl_2_·H_2_O (795mg/l), NaH_2_PO_4_·2H_2_O (690mg/l), Na_2_S·9H_2_O (5mg/l), Urea (1000mg/l), and 20cc of distilled water was used in each experimental group. Also, a solution containing 20cc of modified Fusayama artificial saliva and 20cc of distilled water (with no toothpaste) was used in the control group [3]. Each tooth was brushed in 65cc of its designated solution at the 150-cycle/minute frequency for 4.5 minutes (an equivalent of one month of tooth brushing of each dental surface for 3 times per day). Next, each tooth was rinsed with 20cc of distilled water for 20 seconds, and in order to avoid bracket distortion, the brackets were separated from the teeth using a bur mounted on a handpiece (the brackets and wires were covered with a sterile gauze in this stage). The frictional force was measured using a universal testing machine (Zwick/Roell Z050, Germany).

**Fig. 2:**
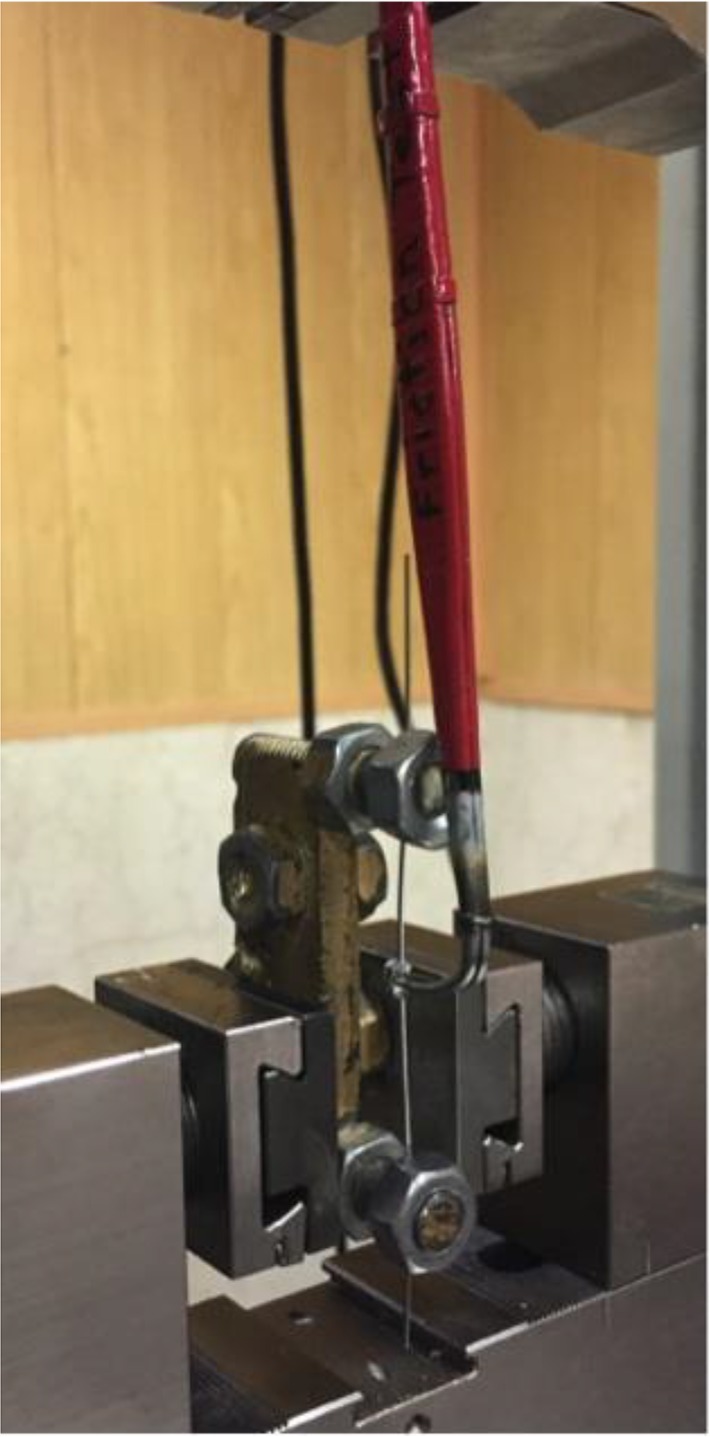
Friction testing apparatus

A custom-made fixture was designed for holding the wires as shown in Figure 2. A plumb line was suspended in order to ensure that the bracket mount was parallel to the vertical line scribed on the steel bar base of the bracket mount set. The load measuring cell was calibrated between 0 and 5 N, and a 5-mm section of the archwire was driven through the bracket at a crosshead speed of 10 mm/minute. Note that no torsion must be generated in the specimen during clamping. The static friction was recorded as the maximum frictional force needed to initiate the movement of the bracket over the 5-mm test distance. The bracket-wire combination was removed after performing each test and a new set was placed. The recorded data were analyzed using one-way analysis of variance (ANOVA). To determine the differences between the groups, Tamhane’s T2 post hoc test was used due to the significant differences between the variances as analyzed by Levene’s test (p<0.005). Statistical analysis was performed using SPSS statistical software (SPSS 22 for Windows; SPSS Inc., Chicago, IL, USA) at the 0.05 significance level.

## RESULTS

Table 2 shows the descriptive data of the frictional resistance values of stainless steel brackets and wires in the experimental and control groups after brushing. The maximum frictional resistance value was related to Colgate® Total® Pro Gum Health toothpaste. The frictional resistance values related to Ortho.Kin®, GUM® Ortho and Colgate® Total® Advanced Whitening toothpastes were not significantly different from that of the control group (P>0.22). However, there was a significant difference in the frictional resistance between Colgate® Total® Pro Gum Health and Colgate® Anticavity toothpastes with that of the control group (P=0.004 and 0.001, respectively).

**Table 2.** The frictional resistance values (N/mm2) between the stainless steel wires and brackets in the experimental and control groups

**Groups**	**Number**	**Minimum**	**Maximum**	**Mean**	**Std. Deviation**	P-value^*^
Control	15	0.078	0.226	0.138	0.041	
Colgate® Total® Advanced Whitening	15	0.084	0.285	0.182	0.052	0.220
Ortho.Kin®	15	0.100	0.205	0.140	0.034	1.000
GUM® Ortho	15	0.087	0.212	0.142	0.033	1.000
Colgate® Total® Pro Gum Health	15	0.123	0.385	0.242	0.083	0.004
Colgate® Anticavity	15	0.154	0.254	0.200	0.032	0.001

* Compared with the control group

## DISCUSSION

Determining the precise amount of the friction produced during orthodontic treatment is difficult due to the diversity of the influencing factors. Several factors may directly or indirectly influence the friction between the wire and bracket such as the alloy type, shape and diameter of orthodontic wires, type of ligation, manufacturing method (sintering vs. casting) and biological factors including saliva and acquired pellicle and plaque [16].

The effect of different types of toothpaste on the frictional resistance between orthodontic metal brackets and archwires has not been previously studied. In the present study, the effect of different types of toothpaste on the frictional resistance between orthodontic stainless steel brackets and archwires was investigated.

The static friction was assessed in this study because it has been considered to be more important than the kinetic friction. The sliding motion of the teeth along an archwire occurs in a series of short steps instead of a continuous movement. Thus, the static friction must be overcome each time the teeth move [17]. In the present study, the ligation method between the bracket and wire was standardized to eliminate the effect of this variable, since the ligation system is one of the variables influencing the frictional force [18].

The results of this study showed that the frictional resistance values related to the orthodontic toothpastes (Ortho.Kin® and GUM® Ortho) were similar to that of the control group. The common material used in both GUM® Ortho and Ortho.Kin® toothpastes is cetylpyridinium chloride (CPC). This ingredient might have been responsible for the decreased frictional resistance of these groups. On the other hand, the highest amount of frictional resistance was related to Colgate® Total® Pro Gum Health toothpaste. This might be due to the presence of Triclosan active component in this toothpaste. However, it should be noted that we did not find supporting data in the literature to confirm the above conclusions and thus, further studies are required in this regard.

It should be noted that the mean frictional resistance value related to Colgate® Total® Advanced Whitening toothpaste was higher than those related to the orthodontic toothpastes and control group even though the difference in the friction between these groups was not significant. Several studies have evaluated the abrasive effect of toothpastes on the enamel. Moghareh-Abed et al [19] and Yaghini et al [20] have investigated the effect of different commercial toothpastes on enamel abrasion. They concluded that there were no significant differences in the enamel abrasion between the evaluated toothpastes.

Furthermore, contradictory information exists about the relationship between the friction and surface roughness. Nishio et al [18] reported that the stainless steel wire with the smoothest surface demonstrated the minimum frictional force value. Saunders and Kusy [21] have shown that the archwire alloy might have a more prominent impact on the frictional characteristics than the bracket type and surface roughness. In addition, Prososki et al [22] and Doshi and Bhad-Patil [23] found no relation between wire roughness and frictional resistance.

In the present study, the frictional resistance values related to Colgate® Total® Advanced Whitening, Colgate® Total® Pro Gum Health, and Colgate® Anticavity toothpastes were approximately similar and higher than that of the control group. Therefore, if a correlation exists between the friction and surface roughness, our results would be coordinated with those of other related studies in which no significant difference was observed in enamel abrasion between the evaluated toothpastes [19,20]. Also, the fluoride contents of the evaluated toothpastes were approximately equal (1450 part per million (ppm) fluoride except for GUM® Ortho with 1490 ppm). Therefore, it may be concluded that fluoride does not have a significant effect on the frictional resistance. Finally, it should be noted that several intraoral variables such as saliva, plaque, chewing force, bone density, tooth number, anatomic configuration, and occlusion can influence the frictional force levels. These factors were not evaluated in the present study; therefore, the frictional forces reported in the current study might be different from the actual forces exerted during orthodontic movements [5]. Further clinical studies are required to investigate the effect of different types of toothpaste on other types of archwires and also on the friction during orthodontic treatments.

## CONCLUSION

According to the results, orthodontic toothpastes did not increase the frictional resistance between the orthodontic stainless steel brackets and wires and can be recommended to patients during orthodontic treatments.
